# Population Structure Analysis Reveals the Rich Genetic Diversity of Honeybee (*Apis mellifera* L.) Populations in Kazakhstan

**DOI:** 10.3390/insects17030318

**Published:** 2026-03-16

**Authors:** Kairat Dossybayev, Aidar Tapelov, Ulzhan Nuraliyeva, Gaukhar Moldakhmetova, Tilek Kapassuly, Altynay Kozhakhmet, Oleg Krupskiy, Merey Torekhanov, Akbota Taufikh, Daryn Bekman, Daniya Ualiyeva, Szilvia Kusza, Makpal Amandykova, Bakytzhan Bekmanov

**Affiliations:** 1 Kazakh Research Institute of Animal Husbandry and Fodder Production, 51 Zhandosov Street, Almaty 050071, Kazakhstan; kairat1987_11@mail.ru (K.D.); tapelov.aidar@gmail.com (A.T.); nua.ulgan@mail.ru (U.N.); gosha_86kz@mail.ru (G.M.); tilek.kapas@mail.ru (T.K.); altynaitg@gmail.com (A.K.); apicenter2000@mail.ru (O.K.); merei_torehanov@mail.ru (M.T.); 05021996@list.ru (A.T.); 2 Faculty of Biology and Biotechnology, Al-Farabi Kazakh National University, 71 Al-Farabi Avenue, Almaty 050040, Kazakhstan; 3 Institute of Genetics and Physiology, 93 Al-Farabi Avenue, Almaty 050060, Kazakhstan; boitube1098@gmail.com (D.B.); daniya.2010@mail.ru (D.U.); 4 Faculty of Veterinary and Zooengineering, Kazakh National Agrarian Research University, 8 Abay Avenue, Almaty 050010, Kazakhstan; 5 Centre for Agricultural Genomics and Biotechnology, University of Debrecen, 4032 Debrecen, Hungary; kusza@agr.unideb.hu

**Keywords:** admixture, *Apis mellifera*, honeybee, genetic diversity, Kazakhstan, mitochondrial DNA, population structure, STR markers

## Abstract

Honeybee (*Apis mellifera* L.) populations are a vital resource for pollination and honey production, yet their genetic diversity in Central Asia remains poorly understood. This study provides a comprehensive genetic assessment of 16 honeybee populations from Kazakhstan, with comparative samples from Russia, Georgia and Kyrgyzstan, utilizing mitochondrial *COI*–*COII* intergenic region and 12 highly polymorphic nuclear STR markers. Mitochondrial DNA analysis revealed the predominance of the Eastern European C lineage (*A. m. carnica*), while a few populations from East Kazakhstan and Russia attributed the M lineage (*A. m. mellifera*), indicating local introgression and the persistence of relict lineages. STR analyses showed high levels of polymorphism and genetic diversity, with variation in heterozygosity and inbreeding across populations. Analyses of population genetic structure delineated four principal genetic clusters shaped by regional differentiation, historical gene flow, and sporadic admixture. Concordance between mitochondrial and nuclear markers confirms the robustness of these findings. Overall, this study highlights the rich genetic diversity of honeybees from Kazakhstan and emphasizes the importance of conserving local populations and implementing selective breeding programs to sustain adaptive potential and long-term apiculture.

## Simple Summary

This study examined the genetic diversity and structure of honeybee (*Apis mellifera* L.) populations from Kazakhstan, with comparison to samples from Kyrgyzstan, Russia and Georgia. By analyzing both mitochondrial DNA (*COI*–*COII* region) and nuclear STR markers, we found that most Kazakhstani populations belong to the Eastern European C lineage, while a few samples show influence from the Western/Northern European M lineage. Our results revealed four main genetic clusters, reflecting regional differentiation, historical gene flow and occasional admixture. These findings highlight the high genetic diversity of honeybees in the region and underscore the importance of conserving local populations for breeding, adaptation and sustainable apiculture.

## 1. Introduction

The western honeybee (*Apis mellifera* L.) is a key pollinator of crops and wild plants worldwide and plays a crucial role in global food security and ecosystem health. In addition to their ecological importance, honeybees are of considerable economic value due to honey and other hive products. However, honeybee populations are increasingly threatened by habitat loss, diseases, climate change and the introduction of non-native subspecies, which can lead to genetic homogenization and reduced local adaptation.

Kazakhstan, with its diverse climates and landscapes, harbors distinct honeybee populations adapted to regional environmental conditions. According to data available as of 2025, Kazakhstan has approximately 12,000 registered beekeepers, and annual honey production reaches about 3900 tons, of which 222.7 tons are exported to both neighboring and more distant countries [1]. The principal honey-producing regions include East Kazakhstan, Pavlodar, Almaty, and Turkestan. Apiculture in the country is largely based on Carpathian and Central Russian honey bee subspecies, while nomadic (migratory) beekeeping practices are predominant in Northern, Southern, and Western Kazakhstan [2]. Local populations represent valuable genetic resources for sustainable apiculture, breeding programs and the conservation of adaptive traits. Understanding the genetic structure and diversity of honeybee populations is therefore critical for maintaining their resilience and guiding conservation strategies.

Honey bee subspecies of *Apis mellifera* were initially classified into three major evolutionary lineages (A, M, and C) based on morphometric analyses, whereas the application of molecular genetic methods subsequently refined this classification and revealed five principal lineages (A, including sub-lineage Z, M, C, O, and Y) [3]. Thus, although enzymatic methods, alongside morphometric approaches, have also been successfully applied to lineage identification in honey bees [4], molecular genetic methods provide substantially higher resolution and are therefore more effective for accurate lineage differentiation. Moreover, morphometric and enzymatic methods do not always yield reliable results, as they are susceptible to variation caused by environmental influences, developmental plasticity, and management practices, which can lead to deviations from standard reference values [5].

Mitochondrial DNA (mtDNA) [6,7], particularly the *COI*–*COII* intergenic region [8,9], is widely used to trace maternal lineages and phylogeographic origins, while nuclear microsatellite (STR) markers provide high-resolution information on intra- and inter-population genetic diversity, population structure, and admixture [10]. Combining mtDNA and STR analyses offers a comprehensive view of honeybee population genetics, capturing both maternal inheritance and nuclear genome variability. Using 22 microsatellite loci, the study by Knoll et al. (2025) [10] assessed the genetic diversity of *Apis mellifera* populations in the Czech Republic through the analysis of worker bees collected from hives and from flowers across 77 districts. Mitochondrial genome data analysis has also been successfully used in honeybee studies. Alsharhi et al. (2025) [11] investigated the genetic diversity and evolutionary relationships of *Apis mellifera jemenitica* across the Arabian Peninsula, Jordan, and Ethiopia using three mtDNA markers (*Cyt b*, *COI*, and *COI*–*COII*) and applying PCR amplification, Sanger sequencing, Maximum-Likelihood phylogenetic analyses, and in silico *DraI COI*–*COII* (DmCC) tests to identify haplotypes and assess intra- and inter-population genetic relationships. In Arkansas, Cleary et al. (2024) [12] characterized mitochondrial DNA variation in *Apis mellifera* by sequencing the *COI*–*COII* intergenic region in 214 colonies and swarms, identifying 23 haplotypes across A, C, M, and O lineages and revealing unique genetic diversity absent from commercial queen breeder stocks. In their study of Turkish honeybee (*Apis mellifera* L.) populations, Özdil et al. (2012) [13] investigated genetic structure across three mitochondrial regions (*16S* rRNA, *COI*, and *ND5*) using PCR amplification, RFLP analysis with 18 restriction enzymes, and direct DNA sequencing to identify nucleotide polymorphisms, novel haplotypes, and phylogenetic relationships among populations. In a comparative study of Western honeybee (*Apis mellifera*) populations, Chen et al. (2022) [14] applied both individual whole-genome sequencing and pooled sequencing to assess genetic diversity and population structure across 14 populations, including 13 subspecies.

Although studies on honeybee genetics have been conducted in Europe, Russia and parts of Central Asia, comprehensive data for Kazakhstan and neighboring regions remain limited. Temirbayeva et al. (2023) [15] investigated the biogeography and potential hybridization of *A. m. pomonella* by analyzing forewing shape variation among local and exotic honeybee populations, providing morphological evidence to distinguish this subspecies and assess introgression from introduced lineages. In addition, the first extensive genetic survey of honeybee populations across 21 regions of Kazakhstan revealed 19 unique mitochondrial *COI*–*COII* haplotypes from the C and M evolutionary lineages and high levels of microsatellite polymorphism, as well as pervasive admixture among subspecies that likely reflect decades of uncontrolled breeding and queen importation practices in the country [16].

Thus, this study aims to fill this gap by characterizing the genetic diversity and population structure of honeybee populations from Kazakhstan, with comparative samples from Russia, Georgia and Kyrgyzstan, using both mtDNA and STR markers. The results provide insights into evolutionary relationships, local adaptation and introgression, offering a foundation for breeding programs and conservation strategies tailored to regional honeybee populations.

## 2. Materials and Methods

### 2.1. Sample Collection

**Figure 1:**
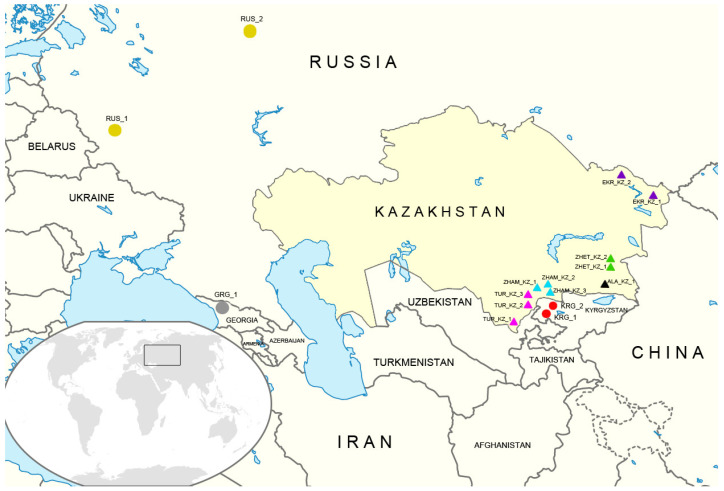
Geographic distribution of honeybee (*Apis mellifera* L.) populations sampled in Kazakhstan and neighboring regions. The coloration of the points demonstrates the population attribution as follows: Almaty region—black; Zhetysu region—green; Zhambyl region—light blue, Turkestan region—purple; Eastern Kazakhstan—violet; Kyrgyzstan—red; Russia—yellow, Georgia—grey. ZHET_KZ_1—*Apis mellifera carnica* from “Kudryashov” apiary, Zhetysu region, Kazakhstan; ZHET_KZ_2—local population from “Koksu” apiary, Zhetysu region, Kazakhstan; ALA_KZ_1—*Apis mellifera carnica* from Almaty region, Kazakhstan; ZHAM_KZ_1—local population from “Manatbek” apiary, Zhambyl region, Kazakhstan; ZHAM_KZ_2—local population from “Bayzhigitov” apiary from Zhambyl region, Kazakhstan; ZHAM_KZ_3—*Apis mellifera carnica* from “Kakalov” apiary, Zhambyl region, Kazakhstan; TRK_KZ_1—*Apis mellifera carnica* from “Kenje” apiary, Turkestan region, Kazakhstan; TRK_KZ_2—*Apis mellifera carnica* from Tulkibas village, Turkestan region, Kazakhstan; TRK_KZ_3—*Apis mellifera carnica* from “Golovashkin” apiary, Turkestan region, Kazakhstan; EKR_KZ_1—*Apis mellifera mellifera* from East Kazakhstan region, Kazakhstan; EKR_KZ_2—local population from Chingistai village, East Kazakhstan region, Kazakhstan; KRG_1—local population from apiary No. 1, Kyrgyzstan; KRG_2—local population from apiary No. 2, Kyrgyzstan; RUS_1—*Apis mellifera mellifera* from Elenka village, Orlov region, Russia; RUS_2—*Apis mellifera mellifera* from Dyakonovo village, Arkhangelsk region, Russia; GRG_1—*Apis mellifera caucasica* from Mukhuri village, Chkhorotsku Municipality, Georgia.

A total of 151 honeybee (*Apis mellifera* L.) samples representing 11 apiaries were collected across diverse regions of Kazakhstan and were considered to represent local populations. In addition, two sites from Kyrgyzstan (30 samples in total) were included in this group as local populations, defined as bee groups that have historically developed within a specific area, are adapted to local environmental conditions (e.g., climate and forage resources), and possess distinct regional characteristics. In contrast, a zoned (regionalized) population refers to a local population that is officially recommended for breeding within a particular region due to its demonstrated adaptability and productivity under those conditions. This concept stands in opposition to non-regionalized or imported subspecies, whose introduction may result in crossbreeding and the erosion of locally adapted traits. Accordingly, two additional populations from the Russian Federation (Dyakonovo and Elenka villages; 19 samples) and one population from Georgia (Mukhuri village; 10 samples) were included in the study as zoned populations [17]. Subspecies classification for each colony was based on beekeeper documentation and records. Moreover, *A. m. mellifera* samples from Chingistai village in the East Kazakhstan Region, a remote mountainous area, were also included. All samples were assigned to populations according to their geographic origin, with subspecies identity documented for each colony. In total, 210 samples from 16 geographically defined populations were analyzed (Figure 1 and Supplementary Table S1). The population codes are provided in Supplementary Table S1 and in the legend of Figure 1, and these codes are used consistently throughout the text of the paper.

### 2.2. DNA Extraction and Quality Control

Genomic DNA was extracted from a single worker bee per colony using the DNeasy Blood & Tissue Kit (Qiagen, Hilden, Germany) from ethanol-preserved specimens and according to the manufacturer’s recommendations. DNA quality and concentration were evaluated using a NanoDrop One C spectrophotometer (Thermo Fisher Scientific, Waltham, MA, USA). Selected samples were further assessed by agarose gel electrophoresis stained with GelRed (Biotium, Fremont, CA, USA), and visualized using an iBright CL1000 imaging system (Thermo Fisher Scientific, Waltham, MA, USA).

### 2.3. Mitochondrial DNA Analysis and Mitotype Characterisation

To characterize the evolutionary lineage and mitochondrial haplotypes, the *COI*–*COII* intergenic region of mitochondrial DNA (mtDNA) was amplified from genomic DNA isolated from honeybees representing 16 different colonies. PCR amplification was performed using PCR Master Mix (Thermo Fisher Scientific, MA, USA) kit and the following primers: E2: 5′-GGC AGA ATA AGT GCA TTG-3′ and H2: 5′-CAA TAT CAT TGA TGA CC-3′ [18]. Reactions were carried out in a Mastercycler Nexus Gradient thermal cycler (Eppendorf, Hamburg, Germany). Amplification products were visualized by electrophoresis in a 1% agarose gel prepared in TAE buffer (0.04 M Tris–acetate, 1 mM EDTA, pH 8.0) and stained with GelRed (Biotium, Fremont, CA, USA). A GeneRuler 100 bp DNA Ladder (Thermo Fisher Scientific, Waltham, MA, USA) was used as a molecular size marker. Following verification of successful amplification, PCR products were purified using the ExoSAP-IT Express PCR Product Cleanup Kit (Thermo Fisher Scientific, Waltham, MA, USA) and sequenced via the Sanger method using the BigDye Terminator v3.1 Cycle Sequencing Kit on a SeqStudio Genetic Analyzer (Applied Biosystems, Thermo Fisher Scientific, Waltham, MA, USA). Resulting electropherograms were manually inspected and edited for base-calling accuracy in BioEdit v.7.7 [19], and sequences were aligned using ClustalX2 [20]. To assign the obtained sequences to the established mitotypes (A, M, C, O, Y) of *Apis* [21,22], we compared novel data with previously published *A. mellifera* sequences available in GenBank (NCBI, Bethesda, MD, USA) following BLAST procedure (https://blast.ncbi.nlm.nih.gov/Blast.cgi, accessed on 14 December 2025). *Bombus terrestris* (JF715210)—closely related but distinct lineage within *Apidae*, were used as an outgroup to root the phylogeny of honeybees *A. mellifera*. *COI*–*COII* haplotypes were additionally determined by performing in silico *Dra*I restriction analysis using Geneious Prime software (Geneious Prime 2025.2 (https://www.geneious.com)). Phylogenetic analysis was performed in MEGA v.11 using the Neighbor-Joining method under the K2P evolutionary model, with 10,000 bootstrap replications [23,24].

### 2.4. Microsatellite Analysis

All honeybee samples were genotyped at twelve highly polymorphic microsatellite loci to assess genetic diversity, population structure, and phylogenetic relationships. Initially, a set of thirty previously validated microsatellite markers was evaluated for allelic polymorphism, reproducibility, and informativeness in *A. mellifera* [25]. Based on this analysis, twelve loci—A007, A024, A028, A043, A088, Ac117, Ap081, Ap243, Ap226, Ap249, SV167, and SV185—were selected for genotyping (Supplementary Table S2). To enable efficient and accurate analysis, the twelve loci were organized into four multiplex PCR panels: panel 1 included A043, SV167, and SV185; panel 2, Ap243, Ac117, and A028; panel 3, Ap226, A007, and Ap249; and panel 4, Ap081, A088, and A024 [26,27].

PCR amplification was performed using PCR Master Mix (Thermo Fisher Scientific, Waltham, MA, USA) kit and primers at optimized concentrations. Amplification success was confirmed by 1.5% agarose gel electrophoresis, and PCR products were purified using the GeneJET PCR Purification Kit (Thermo Fisher Scientific, Waltham, MA, USA). Purified amplicons were diluted to 30 ng/µL for fragment analysis. For capillary electrophoresis, 1 µL of each PCR product was combined with 0.15 µL of GeneScan 600 LIZ Size Standard and 8 µL of Hi-Di Formamide, and the final volume was adjusted to 50 µL with nuclease-free water. Samples were denatured at 95 °C for 4 min and analyzed on a SeqStudio Genetic Analyzer (Applied Biosystems, Thermo Fisher Scientific, Waltham, MA, USA). Allele sizing and calling were performed using GeneMapper v6.0 software, with the GeneScan 600 LIZ serving as an internal size standard.

This multiplex PCR approach, combined with capillary electrophoresis, allowed simultaneous amplification of multiple loci, reducing analysis time and reagent costs while minimizing technical errors. The strategy provided high-resolution, reproducible genotyping and proved highly effective for evaluating intra- and interpopulation genetic variability in honeybee populations [25,26,27].

### 2.5. Structural Analysis of Microsatellite Data

Genotypes were determined based on fluorescence peak profiles using GeneMapper v6.0 (Thermo Fisher Scientific, Waltham, MA, USA) and exported to Microsoft Excel for subsequent processing. Genetic data were analyzed using GenAlEx v6.5 [25]. Principal Coordinate Analysis (PCoA) was performed at the population level based on genetic distance, with only the first and second coordinates (PC1 and PC2) used for graphical representation. Population structure was assessed using STRUCTURE v2.3.4 [28] under an admixture model with correlated allele frequencies and without linkage. Population information was used as a prior. The analysis included a burn-in period of 50,000 iterations followed by 150,000 Markov Chain Monte Carlo (MCMC) iterations. Ten independent runs were conducted for each value of *K* ranging from 1 to 10, each with five replicates per *K*. The optimal number of clusters (*K*) was determined using Evanno’s Δ*K* method [29], as implemented on the CLUMPAK web server [30]. Genetic diversity indices, including the mean number of observed alleles (*Na*), effective alleles (*Ne*), Shannon’s information index (*I*), observed heterozygosity (*Ho*), and expected heterozygosity (*He*), were calculated for each population using GenAlEx v6.5 [25]. Polymorphic information content (PIC) was calculated as the mean expected heterozygosity (He) across loci for each population.

## 3. Results

### 3.1. Genetic Characteristics of the Studied Populations by Mitochondrial DNA

**Figure 2:**
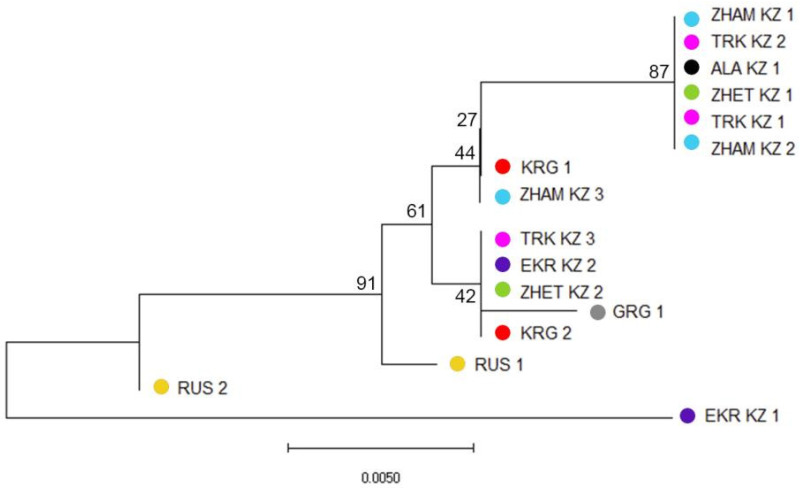
Phylogenetic relationships among honeybee (*Apis mellifera* L.) populations from Kazakhstan, Kyrgyzstan, Russia, and Georgia inferred from sequences of *COI*–*COII* intergenic region. The coloration is in accordance with Figure 1. Bootstrap support is shown next to nodes.

Phylogenetic analysis based on sequence variations in the mitochondrial *COI*–*COII* intergenic region, with 390 bp retained after alignment and trimming of poorly sequenced ends, revealed clear genetic structuring among honeybee populations from Kazakhstan, Kyrgyzstan, Russia, and Georgia (Figure 2).

Most populations clustered according to geographic origin, with several well-supported clades reflecting regional differentiation. The majority of Kazakhstani populations formed closely related groups, indicating shared maternal lineages, although some populations (e.g., different regional samples within ZHAM_KZ, ZHET_KZ, EKR_KZ and TRK_KZ) were separated into distinct subclades, suggesting intra-country mitochondrial diversity. Populations from Kyrgyzstan and Georgia clustered together and showed closer affinity to certain Kazakhstani populations, whereas Russian populations formed distinct branches, reflecting divergence from Central Asian lineages. The observed branch lengths indicate varying levels of genetic differentiation, with some populations exhibiting shallow divergence consistent with recent common ancestry, while others show deeper splits suggestive of historical isolation or distinct maternal origins. Interestingly, one population from Kazakhstan (EKR_KZ_1) clustered closer to the Russian populations, suggesting a shared maternal ancestry or historical gene flow between this population and Russian lineages, possibly reflecting past movements of colonies or the introduction of queens from northern regions.

A comparative analysis was conducted between the sequenced samples obtained in this study and reference sequences from the NCBI (National Center for Biotechnology Information, USA) database. The results are summarized in Supplementary Figures S1 and S2. The phylogenetic tree inferred from sequence variation in the mitochondrial *COI*–*COII* intergenic region revealed clear clustering of the studied populations with established evolutionary lineages of *Apis mellifera*. Most samples from Kazakhstan, Kyrgyzstan, Russia, and Georgia clustered within the C (Eastern European) lineage, showing close relationships with reference sequences of *A. m. carnica* and *A. m. ligustica* from Europe and neighboring regions. Several Kazakhstani populations (e.g., TRK_KZ_1, ALA_KZ_1, TRK_KZ_2, and ZHAM_KZ_2) formed a well-supported subgroup within the C lineage, indicating a shared maternal origin and limited divergence among these populations. In contrast, one Kazakhstani (EKR_KZ_1) and one Russian populations (RUS_2) clustered with reference sequences from the M (Western and Northern European) lineage, including *A. m. mellifera* from Europe, reflecting a distinct maternal ancestry compared with most Central Asian populations. More divergent Kazakhstani populations (e.g., TRK_KZ_3, ZHET_KZ_2, and EKR_KZ_2), together with several zoned populations (GRG_1, KRG_2, and RUS_1), formed a separate cluster basal to the main C-lineage group, indicating increased mitochondrial differentiation and potentially reflecting historical isolation and/or local adaptation. The inclusion of *Apis cerana* and *Bombus sequences* as outgroups clearly separated *A. mellifera* lineages and confirmed the robustness of the phylogenetic reconstruction. Overall, Figure S1 demonstrates that honeybee populations from Kazakhstan and neighboring regions exhibit multiple maternal lineages, with a predominance of the C lineage and evidence of admixture with M lineage, highlighting the complex evolutionary history and genetic heterogeneity of honeybees in Central Asia.

In silico *Dra*I restriction analysis of the *COI*–*COII* sequences was performed using Geneious Prime, and haplotypes were assigned following the criteria described by Chávez-Galarza et al. (2017) [31], with particular emphasis on the internal fragment structure of the restriction profiles (Supplementary Table S3 and Supplementary Figure S3). Based on this approach, samples with the/40/64/restriction pattern were classified as haplotype C2 (ZHET_KZ_2, ALA_KZ_1, ZHAM_KZ_1, ZHAM_KZ_2, TRK_KZ_1, TRK_KZ_2, EKR_KZ_2). Two variants with restriction profiles 113/66/109/65/351 and 113/66/109/65/302 (EKR_KZ_1 and RUS_2, accordingly) showed fragment structures similar to the M haplotypes and were therefore tentatively assigned to the M haplogroup. This interpretation is supported by the phylogenetic analysis (Figures S1 and S2), in which the corresponding samples clustered with M-lineage sequences from the NCBI database. In addition, other restriction profiles did not correspond to any previously described *Dra*I patterns. These profiles were confirmed by re-examining the original sequence data and are therefore presented without haplotype designations (marked as “–”), as they may potentially represent previously undescribed haplotypes.

### 3.2. Genetic Diversity and Variability Analysis

Genetic diversity was analyzed in 16 honeybee populations from Kazakhstan, Kyrgyzstan, Russia and Georgia using several indices: average number of alleles (*Na*), the effective number of alleles (*Ne*), and the Shannon index (*I*), expected (*He*) and observed (*Ho*) heterozygosity, and Polymorphism Information Content (PIC) (Table 1).

The mean number of alleles per locus (*Na*) varied among populations, ranging from the lowest value in RUS_1 (2.667 ± 0.310) to the highest in ZHET_KZ_1 (5.000 ± 0.408), indicating substantial differences in allelic richness across regions. A similar pattern was observed for the effective number of alleles (*Ne*), with values spanning from 1.866 ± 0.177 in ZHET_KZ_2 to 2.747 ± 0.387 in ALA_KZ_1, reflecting variation in allele frequency distributions among populations. Shannon’s information index (*I*) showed moderate levels of genetic diversity overall, with the highest values detected in ZHET_KZ_1 (1.167 ± 0.074), TRK_KZ_1 (1.066 ± 0.147), and EKR_KZ_1 (1.036 ± 0.131), while the lowest diversity was observed in GRG_1 (0.678 ± 0.148) and KRG_2 (0.680 ± 0.146). Expected heterozygosity (*He*) ranged from 0.378 ± 0.074 in KRG_2 and 0.380 ± 0.077 in GRG_1 to 0.597 ± 0.034 in ZHET_KZ_1 and 0.562 ± 0.057 in EKR_KZ_1, suggesting generally moderate genetic variability across the studied populations. Observed heterozygosity (*Ho*) values were typically slightly lower than expected heterozygosity, varying from 0.283 ± 0.094 in GRG_1 to 0.594 ± 0.064 in EKR_KZ_1. Overall, the results demonstrate pronounced heterogeneity in genetic diversity among honeybee populations from Kazakhstan and neighboring regions, with certain populations (e.g., ZHET_KZ_1, EKR_KZ_1, and ALA_KZ_1) exhibiting comparatively higher genetic diversity, while others (e.g., GRG_1 and KRG_2) show reduced variability. PIC values ranged from 0.357 ± 0.171 (ZHET_KZ_2) to 0.552 ± 0.112 (ZHET_KZ_1) across populations.

**Figure 3:**
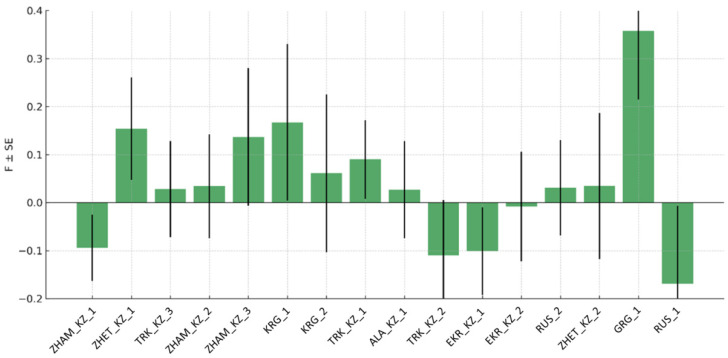
Inbreeding coefficient (*F*) of honeybee (*Apis mellifera* L.) populations from Kazakhstan, Kyrgyzstan, Russia, and Georgia based on 12 microsatellite loci.

Analysis of the inbreeding coefficient (*F*), which reflects the degree of genetic relatedness and homozygosity within populations, revealed considerable variability among the studied groups (Figure 3).

Several populations showed positive *F* values, indicating a deficit of heterozygotes and potential inbreeding or population substructure. The highest positive *F* value was observed in the GRG_1 population, suggesting pronounced homozygosity, while moderately elevated *F* values were also detected in ZHET_KZ_1, ZHAM_KZ_3, KRG_1, and TRK_KZ_1. In contrast, negative *F* values were recorded in multiple populations, including ZHAM_KZ_1, TRK_KZ_2, EKR_KZ_1, EKR_KZ_2, and RUS_1, indicating an excess of heterozygotes that may reflect gene flow, admixture, or outbreeding. Several populations (e.g., TRK_KZ_3, ZHAM_KZ_2, ALA_KZ_1, RUS_2, and ZHET_KZ_2) exhibited *F* values close to zero. Overall, the wide range of *F* estimates across populations highlights heterogeneous demographic histories and mating patterns among honeybee populations from Kazakhstan and neighboring regions, with evidence for both localized inbreeding and enhanced genetic mixing depending on the population.

### 3.3. Genetic Structure Analysis

**Figure 4:**
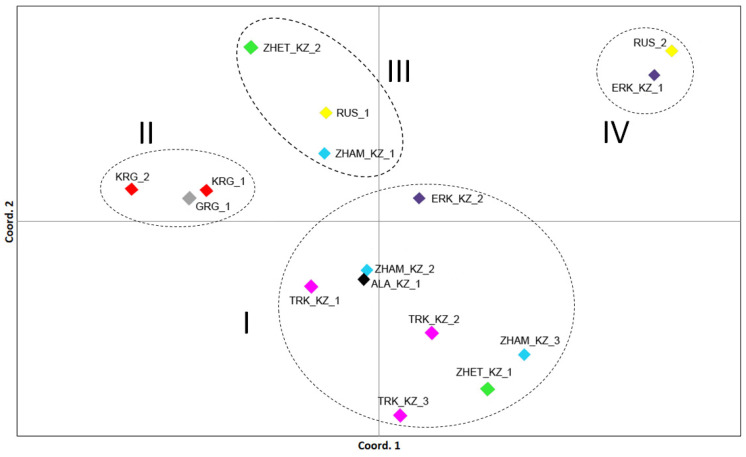
Principal Coordinate Analysis (PCoA) of 16 populations of honeybee (*Apis mellifera* L.) from Kazakhstan, Kyrgyzstan, Russia, and Georgia based on 12 microsatellite loci. The coloration is in accordance with Figure 1.

Principal Coordinate Analysis (PCoA) results were consistent with phylogenetic analysis result highlighting the genetic structure among the studied honeybee populations complimented by their geographic distribution (Figure 4 and Figure S4).

Notably, comparable patterns were observed in the mtDNA analyses (Figure 2 and Figure S1), corroborating this finding. Overall, the principal components analysis at the population level revealed four main genetic clusters: (I) a central Kazakhstani cluster comprising the majority of studied apiaries, (II) a distinct block of Kyrgyzstan and Georgian populations, (III) distant populations, including populations from RUS_1, ZHET_KZ_2 and ZHAM_KZ_1, representing distinct genetic lineages, and (IV) distinct cluster consisting of RUS_2 and EKR_KZ_1 populations. These results indicate that, despite the overall genetic similarity among most Kazakhstani populations, local and distinct lineages exist in the region, offering valuable potential for breeding programs and conservation of genetic diversity.

**Figure 5:**
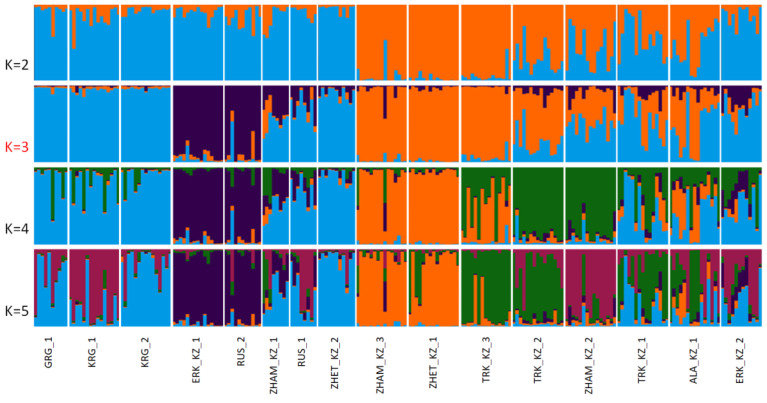
Bayesian clustering of 16 honeybee (*Apis mellifera* L.) populations from Kazakhstan, Kyrgyzstan, Russia, and Georgia based on 12 microsatellite loci, inferred using STRUCTURE.

In addition to PCoA, a Bayesian clustering analysis was conducted using the STRUCTURE software. This approach allows the identification of genetic clusters without a priori assignment of individuals to populations, revealing underlying population structure based on probabilistic allele distributions. STRUCTURE analysis determines the number of genetically distinct clusters (*K*) and quantifies their contributions to the composition of the studied populations. Comparing the results of PCoA and STRUCTURE provides a comprehensive assessment of population structure, capturing both overt and cryptic processes such as migration, introgression, or hybridization among populations. The results of the Bayesian clustering analysis are presented in Figure 5.

**Figure 6:**
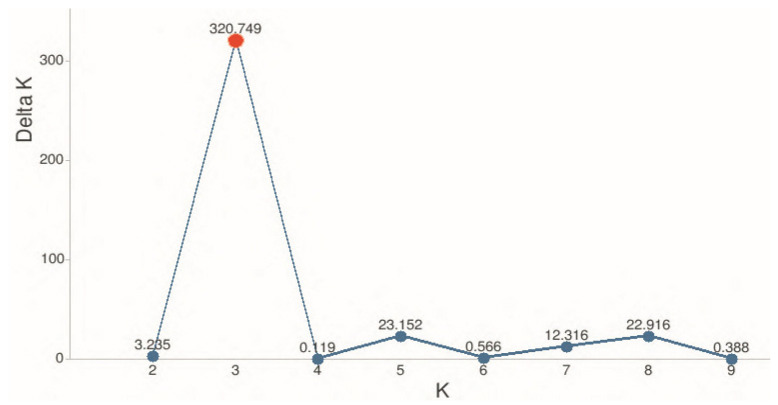
Determination of the optimal number of genetic clusters (K) for 16 honeybee (*Apis mellifera* L.) populations from Kazakhstan, Kyrgyzstan, Russia, and Georgia using Evanno’s method. The ΔK plot shows a pronounced peak at K = 3, indicating the presence of three genetically distinct clusters among the studied populations.

The STRUCTURE analysis confirmed the patterns observed in the population-level analyses (Figure 5). The analysis was conducted for *K* values ranging from 1 to 10, with five repetitions for each value. The model settings included a no-admixture linkage-free model, preliminary use of population information, 50,000 Burn-in iterations, and 150,000 MCMC iterations. The optimal *K* was determined using Evanno’s method [26], which revealed a pronounced peak in the Δ*K* plot at *K* = 3 (Figure 6).

At the optimal number of clusters (K = 3), the STRUCTURE analysis revealed three major genetic components contributing to the studied honeybee populations, indicating pronounced population structure accompanied by varying degrees of admixture (Figure 6). These three clusters broadly correspond to distinct genetic backgrounds rather than strictly mirroring geographic boundaries, suggesting historical gene flow and human-mediated movements of colonies. Several populations showed a clear predominance of a single genetic cluster, reflecting relative genetic homogeneity. In particular, some Kazakhstani populations (e.g., ZHAM_KZ_3, ZHET_KZ_1, TRK_KZ_2, and TRK_KZ_3) were largely dominated by one cluster (orange), indicating limited recent admixture and a more conserved genetic background. Similarly, the GRG_1, KRG_1, KRG_2, EKR_KZ_2, and ZHET_KZ_2 populations exhibited a relatively uniform ancestry profile (blue cluster), suggesting a shared genetic composition. In contrast, other populations displayed pronounced admixture among the three clusters. The ZHAM_KZ_2, ALA_KZ_1 and TRK_KZ_1 populations, as well as the RUS_1 population, exhibited mixed ancestry proportions, indicating ongoing or historical gene flow among genetic clusters. In contrast, the second Russian population (RUS_2) was predominantly assigned to the purple cluster, a pattern also observed in the EKR_KZ_1 population, confirming their close genetic similarity as previously revealed by the PCoA analysis.

## 4. Discussion

Genetic diversity within *Apis mellifera* is increasingly recognized as essential for sustainable apiculture and long-term resilience under environmental change. Across Europe, conservation programs have focused on protecting native subspecies threatened by introgression from commercially favored stocks (De la Rúa et al., 2009; Meixner et al., 2010) [32,33]. In contrast, Central Asia remains comparatively understudied despite its geographic position between major Eurasian evolutionary lineages.

In the present study we provide a comprehensive assessment of the genetic diversity and population structure of honeybee (*Apis mellifera* L.) populations from Kazakhstan and neighboring regions using complementary mitochondrial and microsatellite markers. By integrating mtDNA *COI*–*COII* sequence analysis with STR genotyping, this study offers new insights into maternal lineages, nuclear genetic diversity, and population structure in a region that remains underrepresented in honeybee genetic research.

### 4.1. Maternal Lineages and Phylogeographic Patterns Inferred from mtDNA

Genome-wide analyses have demonstrated that Eurasian honeybee populations have undergone repeated range expansions and secondary contacts (Whitfield et al., 2006; Wallberg et al., 2014) [34,35], and our results suggest that Kazakhstan may represent one such zone of lineage interaction.

Analysis of the mitochondrial *COI*–*COII* intergenic region revealed pronounced phylogeographic structuring among the studied populations, with most samples clustering within the C (Eastern European) lineage. This predominance of the C lineage across Kazakhstan, Kyrgyzstan, and Georgia is consistent with previous reports highlighting the widespread distribution of C-lineage honeybees in Eurasia [3,4,5,6,7,8,9,10,11,12,13,14,15,16,17,18,19,20,21,22,23,24,25,26,27,28,29,30,31,32,33,34,35,36,37,38,39] and in Kazakhstan itself [16] often associated with historical expansion and modern apicultural practices involving *A. m. carnica* and *A. m. ligustica* queens. The close clustering of several Kazakhstani populations (e.g., ZHAM_KZ_1, TRK_KZ_1, ZHET_KZ_1 and others) suggests shared maternal ancestry and relatively recent divergence, likely facilitated by both natural dispersal and human-mediated movements of colonies. The detection of M-lineage haplotypes in RUS_2 and EKR_KZ_1 populations, clustering with *A. m. mellifera* reference sequences, further highlights the coexistence of multiple maternal lineages within the broader study area which is consistent the with the results of [40].

The in silico *Dra*I restriction analysis further supported the mitochondrial lineage assignments inferred from *COI*–*COII* sequence variation. Most samples corresponded to the C2 haplotype, indicating the predominance of the C evolutionary lineage in the studied populations, which is consistent with previous reports describing the widespread distribution of C-lineage honeybees across Eurasia. At the same time, two variants showing restriction profiles similar to the M haplotype were tentatively assigned to the M haplogroup. The clustering of these samples with M-lineage sequences in the phylogenetic analysis supports this interpretation and suggests the presence of M-lineage maternal ancestry within the studied region. Notably, one of these samples originated from the Russian population, which is consistent with previous studies indicating that Russian *Apis mellifera* populations are predominantly associated with the M evolutionary lineage [40]. In addition, several restriction profiles did not correspond to previously described *Dra*I patterns. Although these profiles may potentially represent previously undescribed haplotypes, further confirmation will require additional sampling and analyses, highlighting the need for broader genetic surveys of honeybee populations in Central Asia.

Together, these findings underscore the complex evolutionary history of Central Asian honeybee populations, shaped by both natural biogeographic processes and long-term anthropogenic influence. The identification of more divergent mitochondrial groups among certain Kazakhstani populations (e.g., TRK_KZ_3, EKR_KZ_2 and ZHET_KZ_2) suggests historical isolation or local adaptation, potentially driven by ecological heterogeneity and regional climatic conditions, which are known to play a key role in shaping the distribution and evolutionary trajectories of honeybee populations [41].

### 4.2. Nuclear Genetic Diversity and Population Differentiation

Microsatellite-based analyses revealed moderate but heterogeneous levels of nuclear genetic diversity across populations. Differences in allelic richness, effective number of alleles, and heterozygosity indicate that demographic history, breeding practices, and gene flow vary substantially among regions. Populations such as ZHET_KZ_1, EKR_KZ_1, and ALA_KZ_1 exhibited comparatively high genetic diversity, suggesting larger effective population sizes or greater admixture, whereas reduced diversity in GRG_1 and KRG_2 may reflect founder effects, genetic drift or limited gene flow [42]. The generally lower observed heterozygosity compared with expected heterozygosity in several populations points to deviations from Hardy–Weinberg equilibrium. Such deviations are common in managed honey bee populations and may arise from non-random mating, subdivision within apiaries, selective breeding practices, or the repeated use of a limited number of queens, as reported for Iranian *Apis mellifera* populations, where significant departures from Hardy–Weinberg equilibrium due to heterozygote deficiency have been observed [43]. Conversely, low inbreeding coefficients detected in some populations (e.g., ZHAM_KZ_3, ZHET_KZ_2, RUS_1, and KRG_1) suggest heterozygote excess, consistent with admixture or recent gene flow between genetically distinct groups. The variability in inbreeding coefficients across populations highlights contrasting demographic and management histories. Populations with high positive *F* values, such as GRG_1, may be at risk of reduced adaptive potential, whereas populations showing near-zero or negative *F* values likely benefit from genetic mixing, as high inbreeding increases homozygosity and can reduce evolutionary potential while gene flow and admixture tend to enhance genetic diversity and fitness [44]. Polymorphic information content (PIC) values ranged from moderate to high across populations, indicating that the microsatellite marker set was sufficiently informative for population genetic analyses. These findings emphasize the importance of region-specific management practices when designing breeding and conservation programs.

### 4.3. Population Structure and Admixture Patterns

PCoA analysis and Bayesian clustering (STRUCTURE) consistently revealed partial but non-random population structure, likely reflecting a combination of natural dispersal, historical connectivity, and beekeeping practices, including the introduction and exchange of queens across regions. Notably, differentiation among populations was not absolute, as most groups retained genetic contributions from at least two clusters. The concordance between STRUCTURE and PCoA analyses reinforces the robustness of the inferred population structure and highlights the complex genetic relationships shaped by migration, introgression, and anthropogenic influences. Overall, STRUCTURE analysis at K = 3 indicates that honey bee populations from Kazakhstan and neighboring regions are organized into three primary genetic groups with varying degrees of admixture, underscoring the importance of considering both local conservation efforts and the impact of managed bee movements on regional genetic diversity. Similar patterns have been reported in European honey bee populations, where population structure and admixture are influenced by both natural lineage divergence and apicultural practices such as queen importation and migratory beekeeping [14,45]. Several populations exhibited relatively homogeneous ancestry profiles, suggesting limited recent admixture and the preservation of distinct genetic backgrounds—a pattern commonly observed in stationary or isolated honey bee populations [45]. In contrast, mixed ancestry in populations such as ZHAM_KZ_2, ALA_KZ_1, TRK_KZ_1, and RUS_1 highlights ongoing or historical gene flow, likely driven by migratory beekeeping practices and queen or colony exchange, which are known to increase admixture and reduce geographic structuring in honey bee populations [46]. The strong genetic similarity between RUS_2 and EKR_KZ_1, consistently detected across both PCoA and STRUCTURE analyses, further supports the role of anthropogenic factors in shaping population connectivity across national borders. The concordance between mitochondrial and nuclear datasets strengthens confidence in the inferred population structure and suggests that both maternal inheritance and biparental nuclear variation contribute to the observed genetic patterns. Importantly, the absence of complete population separation indicates that most groups retain genetic contributions from multiple clusters, reflecting a dynamic balance between differentiation and connectivity. The concordance between mitochondrial and nuclear datasets strengthens confidence in this interpretation and suggests that Kazakhstan represents a managed genetic mosaic rather than a set of isolated evolutionary units.

### 4.4. Implications for Conservation and Breeding

Taken together, our results demonstrate that honeybee populations in Kazakhstan and neighboring regions harbor substantial genetic diversity, structured into multiple maternal lineages and nuclear genetic clusters. While admixture has likely enhanced genetic variability in some populations, it also raises concerns about genetic homogenization and the potential loss of locally adapted genotypes. Populations exhibiting distinct mitochondrial lineages or elevated nuclear diversity represent valuable genetic resources that should be prioritized for conservation. For sustainable apiculture, breeding programs should aim to balance productivity improvements with the preservation of regional genetic diversity. Controlled queen breeding, reduced reliance on imported stock, and the protection of locally adapted populations could help maintain evolutionary potential and resilience in the face of environmental change. Given its geographic position and lineage composition, Central Asia should be considered an important component of broader Eurasian honeybee conservation strategies.

While the combined use of mitochondrial and microsatellite markers provides valuable insights into maternal ancestry and biparental structure, these markers have limitations. Mitochondrial DNA reflects only maternal inheritance, and microsatellites, although informative for assessing diversity and structure, do not capture genome-wide adaptive variation. Future studies employing high-density SNP panels, as applied in global surveys (Wallberg et al., 2014) [35], would enable finer-scale inference of demographic history and local adaptation.

## 5. Conclusions

This study provides the first comprehensive assessment of the genetic diversity and population structure of honeybee (*Apis mellifera* L.) populations from Kazakhstan and neighboring regions using a combined analysis of mitochondrial *COI*–*COII* sequences and nuclear microsatellite markers. Both sequence analysis and in silico *Dra*I restriction profiling confirmed the predominance of the C mitochondrial lineage in most studied populations, with the majority of samples corresponding to the C2 haplotype. At the same time, the detection of variants with restriction profiles similar to the M haplotype indicates the presence of M-lineage maternal ancestry in the region, reflecting a complex evolutionary history influenced by both natural processes and human-mediated movements. Nuclear STR analyses demonstrated moderate but uneven levels of genetic diversity among populations, highlighting regions with high conservation value as well as populations showing signs of reduced variability or inbreeding. PCoA and Bayesian clustering (STRUCTURE) analyses consistently identified three major genetic clusters with varying degrees of admixture, underscoring the presence of structured yet interconnected populations across Central Asia. In addition, several restriction profiles did not correspond to previously described *Dra*I patterns, suggesting the possible presence of previously undescribed haplotypes, although this requires further confirmation through expanded sampling and additional analyses. Together, these findings emphasize the importance of preserving locally adapted honeybee genetic resources, particularly in Kazakhstan, and provide a valuable genetic baseline for the development of targeted breeding programs, conservation strategies, and sustainable management of honeybee populations in the region.

## Figures and Tables

**Table 1:** Genetic diversity parameters of honeybee (*Apis mellifera* L.) populations from Kazakhstan, Kyrgyzstan, Russia, and Georgia based on 12 microsatellite loci. *Na*—observed number of alleles; *Ne*—effective number of alleles; *I*—Shannon’s information index; *He*—expected heterozygosity; *Ho*—observed heterozygosity; PIC—Polymorphism Information Content.

Pop	*Na*	*Ne*	*I*	*He*	*Ho*	PIC
ZHET_KZ_1	5.000 ± 0.408	2.666 ± 0.213	1.167 ± 0.074	0.597 ± 0.034	0.506 ± 0.070	0.552 ± 0.112
ZHET_KZ_2	2.917 ± 0.336	1.866 ± 0.177	0.711 ± 0.102	0.408 ± 0.056	0.409 ± 0.090	0.357 ± 0.171
ALA_KZ_1	4.250 ± 0.605	2.747 ± 0.387	1.029 ± 0.152	0.538 ± 0.070	0.528 ± 0.089	0.536 ± 0.186
ZHAM_KZ_1	3.583 ± 0.434	2.420 ± 0.358	0.936 ± 0.128	0.504 ± 0.056	0.542 ± 0.060	0.458 ± 0.194
ZHAM_KZ_2	3.750 ± 0.372	2.546 ± 0.267	0.991 ± 0.122	0.541 ± 0.061	0.517 ± 0.087	0.490 ± 0.202
ZHAM_KZ_3	3.167 ± 0.322	1.992 ± 0.203	0.770 ± 0.104	0.438 ± 0.057	0.394 ± 0.094	0.387 ± 0.179
TRK_KZ_1	4.667 ± 0.555	2.684 ± 0.355	1.066 ± 0.147	0.535 ± 0.067	0.500 ± 0.072	0.497 ± 0.227
TRK_KZ_2	3.750 ± 0.494	2.375 ± 0.292	0.900 ± 0.147	0.486 ± 0.075	0.544 ± 0.103	0.526 ± 0.143
TRK_KZ_3	3.833 ± 0.548	2.611 ± 0.397	0.957 ± 0.168	0.499 ± 0.081	0.506 ± 0.101	0.551 ± 0.176
EKR_KZ_1	3.917 ± 0.484	2.726 ± 0.329	1.036 ± 0.131	0.562 ± 0.057	0.594 ± 0.064	0.505 ± 0.201
EKR_KZ_2	3.250 ± 0.392	2.267 ± 0.266	0.851 ± 0.137	0.473 ± 0.071	0.451 ± 0.084	0.462 ± 0.198
KRG_1	3.250 ± 0.617	2.272 ± 0.433	0.785 ± 0.162	0.430 ± 0.076	0.422 ± 0.106	0.459 ± 0.191
KRG_2	2.917 ± 0.484	1.957 ± 0.290	0.680 ± 0.146	0.378 ± 0.074	0.328 ± 0.107	0.405 ± 0.201
RUS_1	2.667 ± 0.310	2.068 ± 0.244	0.719 ± 0.125	0.429 ± 0.072	0.500 ± 0.117	0.403 ± 0.195
RUS_2	4.000 ± 0.326	2.389 ± 0.251	0.981 ± 0.097	0.530 ± 0.046	0.508 ± 0.066	0.477 ± 0.155
GRG_1	2.917 ± 0.514	1.919 ± 0.235	0.678 ± 0.148	0.380 ± 0.077	0.283 ± 0.094	0.401 ± 0.203

## Data Availability

The original contributions presented in this study are included in the article. New sequences obtained in this study were deposited to the GenBank under accession numbers PX974352-PX974367 (https://www.ncbi.nlm.nih.gov/).

## Supplementary Materials

The following supporting information can be downloaded at: https://www.mdpi.com/article/10.3390/insects17030318/s1, Table S1: Sample sizes and geographic locations of the 16 honeybee (*Apis mellifera* L.) populations analyzed in the study; Table S2: Microsatellite loci used for genotyping honeybee (*Apis mellifera* L.) populations; Table S3: *COI*–*COII* haplotypes determined by in silico *DraI* restriction analysis using Geneious Prime software. Figure S1: Extended phylogenetic relationships of Kazakhstani honeybees (*Apis mellifera* L.) and other worldwide populations based on GenBank sequences; Figure S2: Detailed phylogenetic analysis of mtDNA COI–COII haplotypes of Kazakhstani honey bee samples and another sequences of the M and C evolutionary lineages from GenBank. The phylogenetic tree was constructed using the Neighbor-Joining method in MEGA11 with 1000 bootstrap replicates. Bootstrap values (%) are shown at the nodes. Figure S3: In silico *DraI* restriction patterns of the mitochondrial *COI*–*COII* region in 16 *Apis mellifera* populations generated using Geneious Prime software. Figure S4: Principal Component Analysis (PCA) of 16 honey bee (*Apis mellifera* L.) populations from Kazakhstan, Kyrgyzstan, Russia, and Georgia based on 12 microsatellite loci.

## Abbreviations

The following abbreviations are used in this manuscript:

RSE	Republican State Enterprise
DNA	Deoxyribonucleic acid
PCoA	Principal Coordinate Analysis
*COI*–*COII*	Cytochrome c oxidase subunit I–cytochrome c oxidase subunit II
