# Supplementary material for: Population Structure Analysis Reveals the Rich Genetic Diversity of Honeybee (Apis mellifera L.) Populations in Kazakhstan
Source: Insects. 2026 Mar 16;17(3):318. doi: 10.3390/insects17030318 (PMC13026694; doi:10.3390/insects17030318)
Supplement: Supplementary file 1 [file insects-17-00318-s001.zip › insects-4174651-supplementary.pdf]

Supplementary Tables

Table S1. Sample sizes and geographic locations of the 16 honeybee (*Apis mellifera* L.) populations analyzed in the study.

№	Code	Geograpic location	Number	Subspecies	Mitotype	North latitude	East longitude
**Local populations**							
1	ZHET_KZ_1	"Kudryashov", Zhetysu region, Kazakhstan	15	*Apis mellifera carnica*	C	44.867431	78.74244
2	ZHET_KZ_2	"Koksu", Zhetysu region, Kazakhstan	11	KZ local population	C	44.710852	79.025248
3	ALA_KZ_1	Almaty region, Kazakhstan	15	*Apis mellifera carnica*	C	43.435683	77.329931
4	ZHAM_KZ_1	"Manatbek", Zhambyl region, Kazakhstan	8	KZ local population	C	42.964760	75.650513
5	ZHAM_KZ_2	"Bayzhigitov", Zhambyl region, Kazakhstan	15	KZ local population	C	42.964985	75.650055
6	ZHAM_KZ_3	"Kakalov", Zhambyl region, Kazakhstan	15	*Apis mellifera carnica*	C	42.968367	71.473569
7	TRK_KZ_1	"Kenje", Turkestan region, Kazakhstan	15	*Apis mellifera carnica*	C	42.513445	70.197256
8	TRK_KZ_2	Tulkibas village, Turkestan region, Kazakhstan	15	*Apis mellifera carnica*	C	42.554851	70.286229
9	TRK_KZ_3	"Golovashkin", Turkestan region, Kazakhstan	15	*Apis mellifera carnica*	C	40.872514	68.256013
10	EKR_KZ_1	East Kazakhstan region, Kazakhstan	15	*Apis mellifera mellifera*	M	50.319843	83.590694
11	EKR_KZ_2	Chingistai village, East Kazakhstan region, Kazakhstan	12	KZ local population	C	49.186311	85.873636
12	KRG_1	Apiary No. 1, Kyrgyzstan	15	KRG local population	C	41.652957	72.743689
13	KRG_2	Apiary No. 2, Kyrgyzstan	15	KRG local population	C	41.524948	73.314088
**Zoned populations**							
14	RUS_1	Elenka village, Smolensk region, Russia	8	*Apis mellifera mellifera*	C	54.873647	34.468927
15	RUS_2	Dyakonovo village, Arkhangelsk region, Russia	11	*Apis mellifera mellifera*	M	61.098351	48.047312
16	GRG_1	Mukhuri village, Chkhorotsku Municipality, Georgia	10	*Apis mellifera caucasica*	C	42.618993	42.149774
		Overall	210				

Table S2. Microsatellite loci used for genotyping honeybee (*Apis mellifera* L.) populations.

№	Name	F (5'-3')	R (3'-5')	Marks (F) (5'-3')
1	A007	CCCTTCCTCTTTCATCTTCC	GTTAGTGCCCTCCTCTTGC	6-FAM
2	A024	CACAAGTTCCAACAATGC	CACATTGAGGATGAGCG	VIC
3	A028	GAAGAGCGTTGGTTGCAGG	GCCGTTCATGGTTACCACG	6-FAM
4	A043	CACCGAAACAAGATGCAAG	CCGCTCATTAAGATATCCG	VIC
5	A088	CGAATTAACCGATTTGTCG	GATCGCAATTATTGAAGGAG	PET
6	Ac117	CGGTTCATCTTCCCTTTATTTC	CCACGGGATTATTATCGTTTATC	6-FAM
7	Ap081	GGATCGTCGAGGCGTTGA	GAAAAGTATTCCGCCGAGCA	6-FAM
8	Ap243	AATGTCCGCGAGCATCTG	TGTTTACGAGAATTCGACGGG	HEX
9	Ap226	AACGGTGTTCGCGAAACG	AGCCAACTCGTGCGGTCA	VIC
10	Ap249	CGCGCGACGACGAAATGT	CAGTCCTTTGATTCGCGCTACC	VIC
11	SV167	GATCTTCGCGATTCTTCGCA	CTGGTTCACACGGGCAGGTA	VIC
12	SV185	AGCTCACGCAGCACATGC	GACGTTGTTTCCATCACCACTC	6-FAM

Table S3. *COI-COII* haplotypes determined by in silico *DraI* restriction analysis using Geneious Prime software.

**Population**	**Country**	**Subspecies**	***DraI* fragments (bp) (*in silico*)**	**Haplogroup**	**Haplotype**
ZHET_KZ_1	Kazakhstan	A.m.carnica	64/80/382	C	-
ZHET_KZ_2	Kazakhstan	KZ local population	10/40/64/404	C	C2
ALA_KZ_1	Kazakhstan	A.m.carnica	10/40/64/403	C	C2
ZHAM_KZ_1	Kazakhstan	KZ local population	10/40/64/404	C	C2
ZHAM_KZ_2	Kazakhstan	KZ local population	10/40/64/404	C	C2
ZHAM_KZ_3	Kazakhstan	A.m.carnica	40/63/389	C	-
TRK_KZ_1	Kazakhstan	A.m.carnica	10/40/64/403	C	C2
TRK_KZ_2	Kazakhstan	A.m.carnica	10/40/64/404	C	C2
TRK_KZ_3	Kazakhstan	A.m.carnica	64/80/383	C	-
EKR_KZ_1	Kazakhstan	A.m.mellifera	113/66/109/65/351	M	-
EKR_KZ_2	Kazakhstan	KZ local population	10/40/64/403	C	C2
KRG_1	Kyrgyzstan	KRG local population	105/404	C	-
KRG_2	Kyrgyzstan	KRG local population	46/64/404	C	-
RUS_1	Russia	A.m.mellifera	53/65/362	C	-
RUS_2	Russia	A.m.mellifera	113/66/109/65/302	M	-
GRG_1	Georgia	A.m.caucasica	42/65/404	C	-

Supplementary Figures

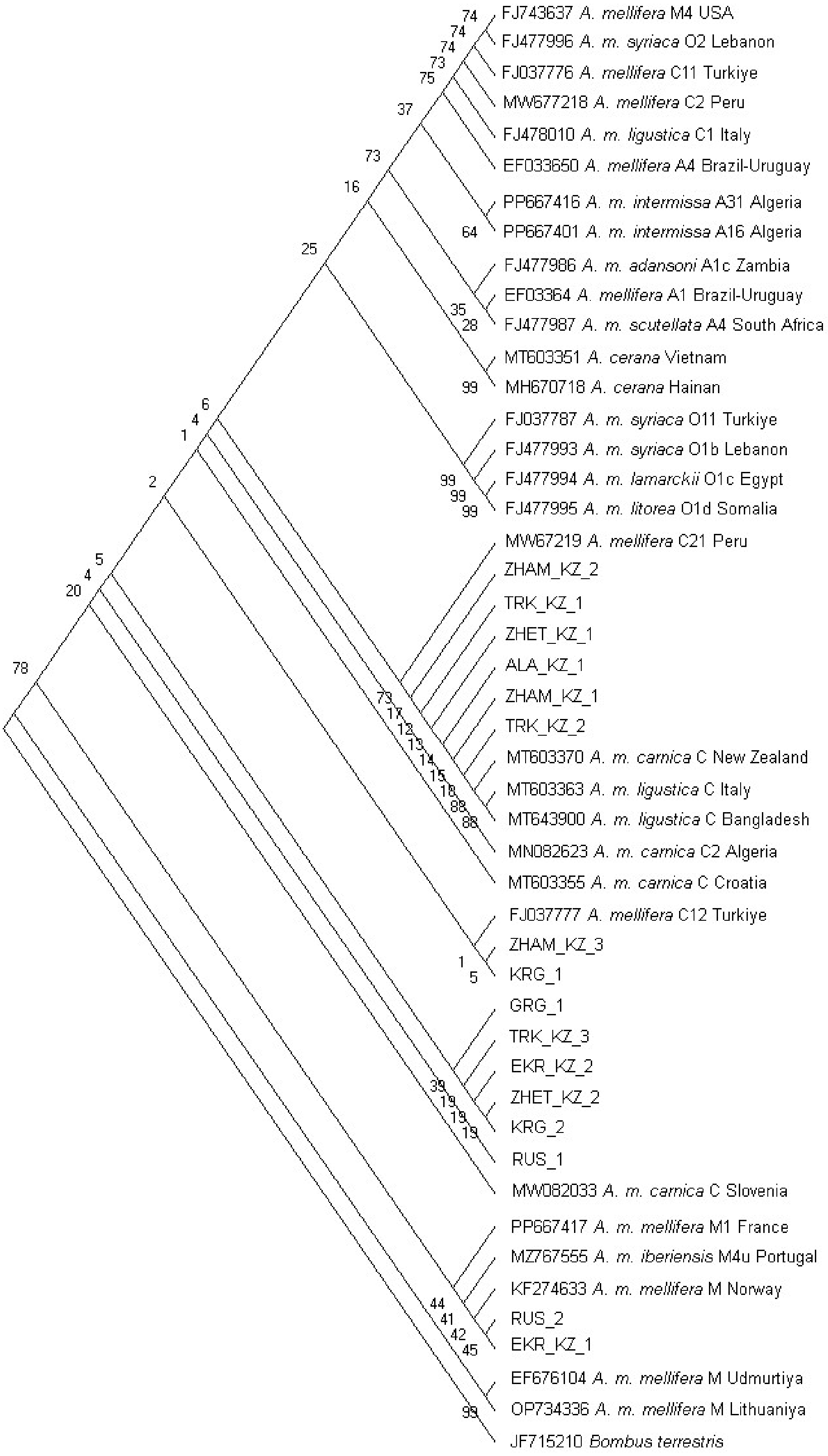

Figure S1. Extended phylogenetic relationships of Kazakhstani honeybees (*Apis mellifera* L.) and other worldwide populations based on GenBank sequences.

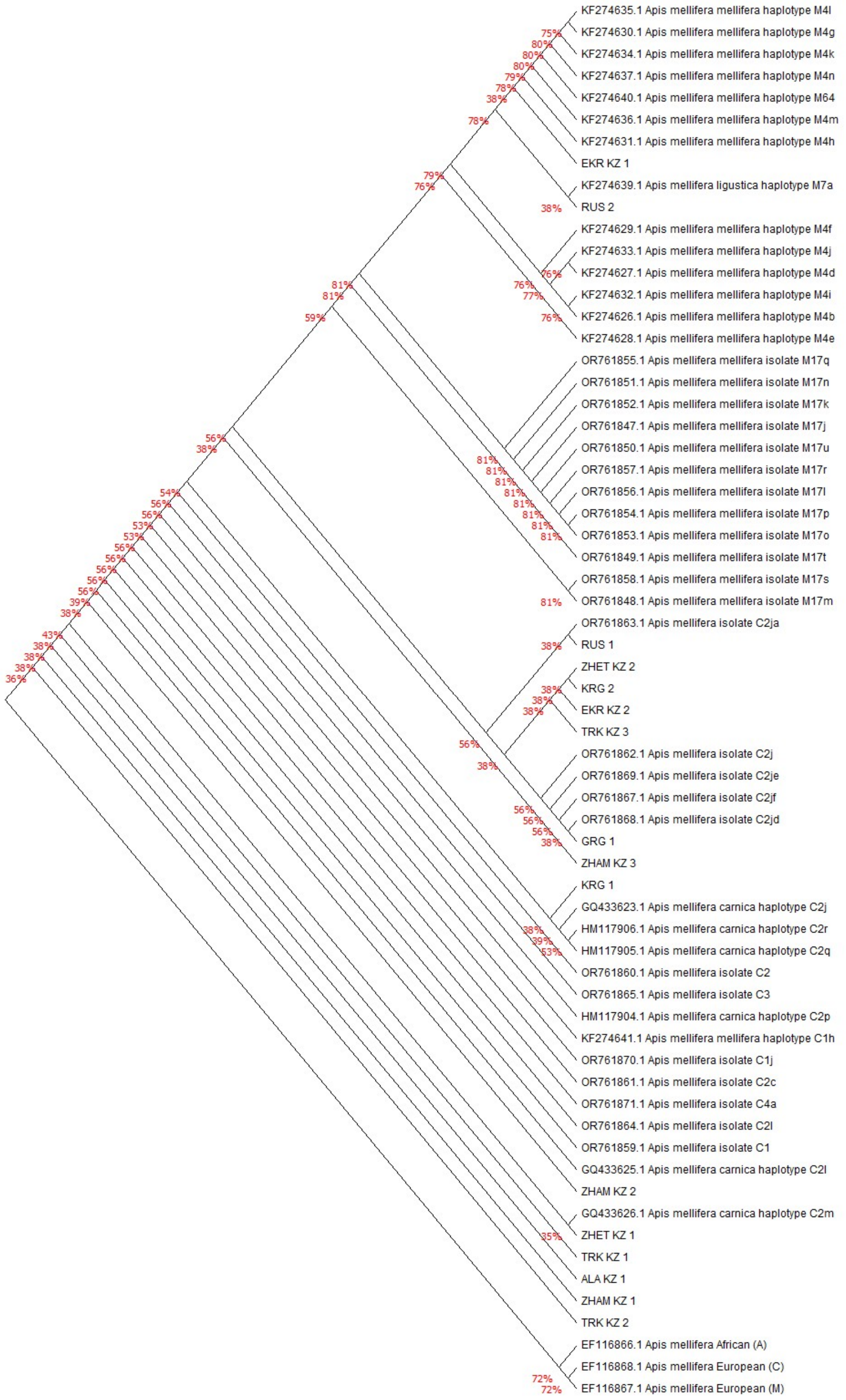

Figure S2. Detailed phylogenetic analysis of mtDNA COI–COII haplotypes of Kazakhstani honey bee samples and another sequences of the M and C evolutionary lineages from GenBank. The phylogenetic tree was constructed using the Neighbor-Joining method in MEGA11 with 1000 bootstrap replicates. Bootstrap values (%) are shown at the nodes.

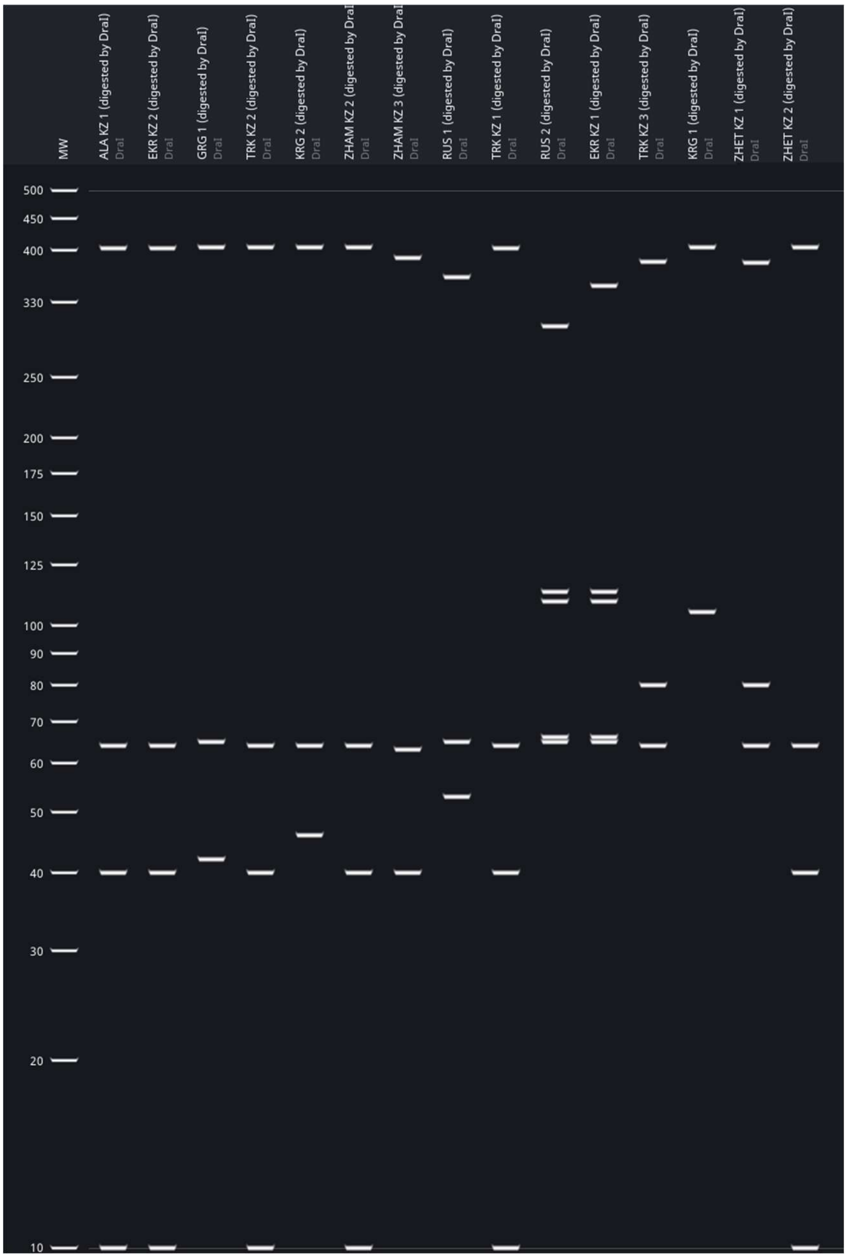

Figure S3. *In silico Dra*I restriction patterns of the mitochondrial *COI–COII* region in 16 *Apis mellifera* populations generated using Geneious Prime software.

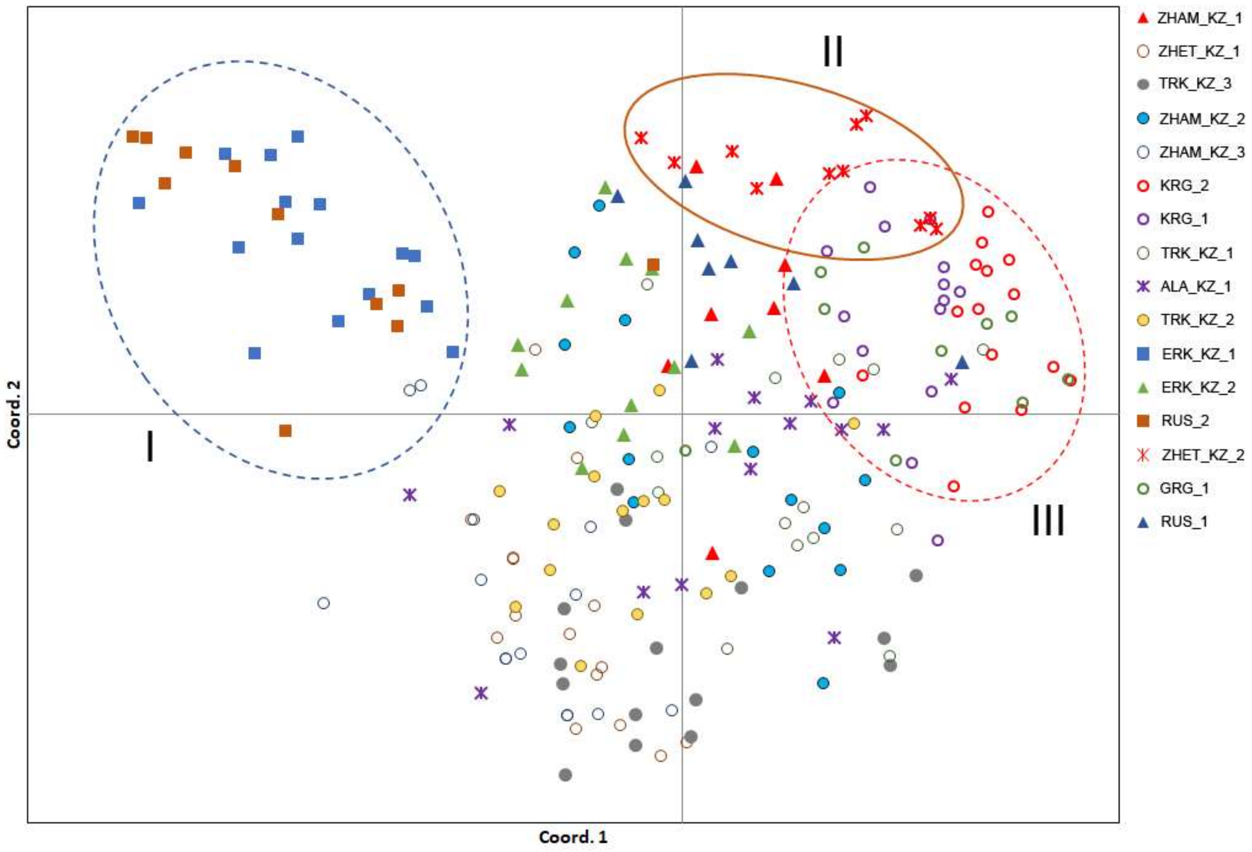

Figure S4. Principal Component Analysis (PCA) of 16 honey bee (*Apis mellifera* L.) populations from Kazakhstan, Kyrgyzstan, Russia, and Georgia based on 12 microsatellite loci.
